# Consciousness emerges from temporal integration across biological scales: from cellular memory to phenomenological experience

**DOI:** 10.3389/fnint.2026.1772467

**Published:** 2026-05-12

**Authors:** Alfredo López Parra

**Affiliations:** Independent Researcher, Mexico City, Mexico

**Keywords:** basal coherence, bioelectric signaling, cellular memory, consciousness, Emergent Flow Theory, Libet paradigm, temporal integration, vagal regulation

## Abstract

Consciousness remains one of the central challenges in contemporary neuroscience, in part due to the absence of an explicit framework describing the temporal constraints required for integrative processing. While influential models such as Integrated Information Theory and the Free Energy Principle characterize structural and functional aspects of conscious systems, they remain largely agnostic regarding the minimum temporal windows necessary for information to become phenomenologically accessible. We propose that temporal integration constitutes a biologically invariant constraint operating across multiple organizational scales. Drawing on recent experimental evidence of memory formation in non-neuronal cells, we introduce the concept of a minimal bioelectrical/biochemical temporal window (ΔT_b) governing cellular information consolidation. We propose that this foundational temporal constraint may contribute to organismic basal coherence (γ) through bioelectric and autonomic mechanisms, which in turn modulate higher-order perceptual and phenomenological integration. Within the Emergent Flow Theory framework, classical findings from the Libet paradigm are reinterpreted not as evidence against agency, but as reflecting necessary delays associated with multilevel temporal integration. By linking cellular memory, bioelectric signaling, vagal–autonomic regulation, and cortical dynamics, this work outlines a unified temporal architecture of conscious processing that is biologically grounded, mechanistically plausible, and empirically testable using multimodal neurophysiological protocols. The proposed pathway linking cellular temporal integration (ΔT_b) to organismic autonomic coherence (γ) is classified as mechanistically plausible, requiring empirical validation within the EFT framework.

## 1 Introduction: the problem of time in consciousness

### 1.1 From cortical cognition to the temporal constraint

Consciousness, commonly defined as the unified and coherent subjective experience of being, remains one of the most persistent challenges in contemporary neuroscience. Despite substantial theoretical and empirical advances, a foundational gap persists in current models: the absence of an explicit account of time as a necessary constraint for conscious integration.

Influential frameworks such as Integrated Information Theory (IIT) (Tononi et al., 2016), which quantifies informational complexity (Φ), and the Free Energy Principle (FEP) (Friston, 2010), which characterizes biological systems as minimizing prediction error through active inference, have provided powerful insights into what is integrated in conscious systems–informational structure, functional coupling, and predictive variables. However, these approaches remain largely agnostic with respect to when, and within what temporal bounds, such integration must occur for a conscious moment to emerge.

Temporal integration cannot be assumed to be continuous, instantaneous, or trivial. On the contrary, time constitutes a fundamental physical and biological constraint on information processing. Any theory of consciousness that omits an explicit temporal architecture risks leaving unexplained how distributed processes are consolidated into a phenomenologically unified present.

### 1.2 The Libet paradigm and the necessity of ΔT

The centrality of time to conscious phenomenology was empirically highlighted by the seminal experiments of Libet (1985). These studies revealed a systematic temporal dissociation between the onset of preconscious neural activity–the Readiness Potential–and the later moment at which subjects reported conscious awareness of intention (the W judgment). This delay, on the order of several hundred milliseconds, demonstrated that conscious access is preceded by a non-negligible interval of unconscious integration.

Rather than undermining the concept of agency, the Libet paradigm underscores a critical point: conscious experience does not arise instantaneously, but depends on a finite temporal window of integration, here denoted as ΔT^1^. Subsequent psychophysical and neurophysiological research has reinforced this conclusion, suggesting that perception itself unfolds within rhythmic or discretized temporal frames rather than as a continuous stream (Pöppel, 1997; VanRullen and Koch, 2003).

Together, these findings compel a reframing of consciousness–not as a static state localized in space, but as an emergent process unfolding over time. The fundamental question therefore shifts from where consciousness occurs to when and over what duration information must be integrated to become phenomenologically accessible. Integration requires a minimal ΔT.

### 1.3 The Emergent Flow Theory and the Temporal Invariance Thesis

The Emergent Flow Theory (EFT) is introduced here as a framework explicitly designed to address this missing temporal architecture. EFT situates consciousness within a hierarchy of physiologically measurable integration windows, proposing that conscious experience emerges from the coordinated integration of information across specific temporal scales.

The core premise of EFT can be summarized as follows: consciousness is not an instantaneous state, but the result of integration processes that require specific temporal windows (ΔT). This dependence on temporal integration constitutes an invariant constraint of adaptive biological systems, from non-neuronal cellular memory (ΔT_b) to human autonomic–cortical integration (ΔT^I^, ΔT_p_, ΔT^f^).

This Temporal Invariance Thesis does not imply that all biological systems possess phenomenological consciousness. Rather, it posits that the requirement of a finite integration window (ΔT) is a necessary but not sufficient condition for any adaptive system capable of consolidating information across time, regardless of its level of organizational complexity.

The aim of the present article is to extend the operational and testable core of EFT–originally formulated at the level of human cortical and autonomic integration–toward its most fundamental biological substrates. By incorporating recent evidence demonstrating non-neuronal cellular memory as a temporally structured process (Kukushkin and Carew, 2017; Kukushkin et al., 2024), we argue that the ΔT operator represents an invariant biological constraint. This constraint provides the molecular and cellular groundwork for basal coherence (γ), which in EFT functions as a necessary enabling condition for conscious integration in the human brain.

## 2 The Emergent Flow Theory (EFT): an operational temporal framework

This section outlines the operational core of the Emergent Flow Theory (EFT), defining consciousness as a temporally constrained integrative process and introducing the hierarchical time windows that structure conscious experience. The goal is to clearly delimit what EFT operationalizes at the neural level and to identify the point at which an extension toward more fundamental biological levels becomes necessary.

### 2.1 Operational core: consciousness as emergent flow

The Emergent Flow Theory (EFT) provides a functional and phenomenological description of consciousness by postulating that it is not a stationary state, but an emergent process resulting from the integration of information across distinct temporal scales. Within EFT, conscious experience arises only when informational, physiological, and temporal conditions jointly converge.

This relationship is formalized through the central EFT heuristic integration relation:


𝒞=γ⋅n⋅α⁢_⁢nk⋅Δ⁢T

Important clarification: this expression is not intended as a dimensionally closed physical law, but as a heuristic systems-level formal relation organizing the jointly necessary constraints on conscious integration. It functions as a formal organizational framework that brings together the key constraints whose joint contribution determines the system’s capacity for conscious integration. This approach parallels other systems-level models in neuroscience (e.g., neural mass models, mean-field approximations) where heuristic formulations guide empirical investigation without requiring first-principles physical derivation.

Importantly, PCI is used here as a partial empirical approximation of the global systems-level variable c rather than as a direct proxy for each local integration coefficient α_nk. Each term captures a necessary–but individually insufficient–component of conscious integration. Table 1 provides explicit units and empirical proxies for each parameter.

**TABLE 1:** Phenomenological parameters of the EFT heuristic integration relation.

Param.	Units/range	Operational definition	Empirical proxy	Tier
*C*	Dimensionless index (0–1)	Global phenomenological index of conscious integration capacity at the systems level	Perturbational Complexity Index (PCI) as operational approximation of global integration capacity (Casali et al., 2013)	Tier-1 established
γ	ms (RMSSD) or ms^2^ (HF-HRV)	Basal autonomic coherence; emergent physiological synchronization indexed by vagal tone	Heart rate variability metrics (HRV: RMSSD, HF power)	Tier-1 established
n	Count (dimensionless)	Active neural units contributing to integration	fMRI BOLD activation extent	Tier-1 established
α_nk	Hz (s^–1^)	Local functional integration coefficient describing efficiency of network coupling across nodes and temporal windows	Gamma-band coherence; effective connectivity; perturbational propagation metrics	Tier-1 established
ΔT	ms (neural); min–hr (ΔT_b)	Temporal integration window; minimum duration for consolidation at each organizational level	ERP latencies (P300, RP); CREB kinetics for ΔT_b	Tier-1 (neural); Tier-2 (ΔT_b)

The EFT expression *C* = γ⋅ n ⋅α_nk ⋅ΔT is a heuristic systems-level formal relation organizing the key variables governing conscious integration, rather than a physically derived equation with strict dimensional consistency. C is a global systems-level index operationally approximated by PCI; it is distinct from the local integration coefficient α_nk, which is approximated by gamma-band coherence and effective connectivity measures. ΔT at the cellular level (ΔT_b) is classified Tier-2 pending empirical validation linking it to γ. HRV, heart rate variability; RMSSD, root mean square of successive RR differences; HF, high-frequency; PCI, Perturbational Complexity Index; RP, Readiness Potential.

γ (Emergent Basal Coherence): represents the system’s baseline physiological and energetic coherence, arising from bottom-up autonomic, interoceptive, and homeostatic processes (e.g., vagal tone, arousal regulation, metabolic stability). γ is not a fixed parameter but an emergent, dynamic property that modulates the system’s sensitivity to information and its readiness for integration.

n (Number of Active Units): denotes the number of functional elements contributing to processing (e.g., neurons, neuronal assemblies, or cortical regions). This parameter captures the scale of participation but does not, by itself, guarantee integration or conscious access.

α_nk (Functional Integration): describes the efficiency and complexity with which active units interact, reflecting the balance between differentiation and integration. Empirically, this term is associated with measures of functional coupling, effective connectivity, and perturbational propagation dynamics, including gamma-band coherence and related TMS–EEG interaction metrics (Casali et al., 2013; Sarasso et al., 2015).

ΔT (Temporal Integration Window): the critical temporal operator of EFT. ΔT represents the minimum duration required for incoming information to be consolidated into a stable, functionally accessible state capable of contributing to subsequent conscious moments.

Throughout this manuscript, claims are classified according to an explicit epistemic tier framework. Tier-1 designates empirically established findings with direct experimental support from multiple independent laboratories. Tier-2 designates mechanistically plausible hypotheses grounded in established physiology but requiring direct empirical validation within the EFT context. Tier-3 designates explicitly speculative theoretical extensions, confined to Supplementary materials.

### 2.2 Established temporal windows in EFT

**FIGURE 1:**
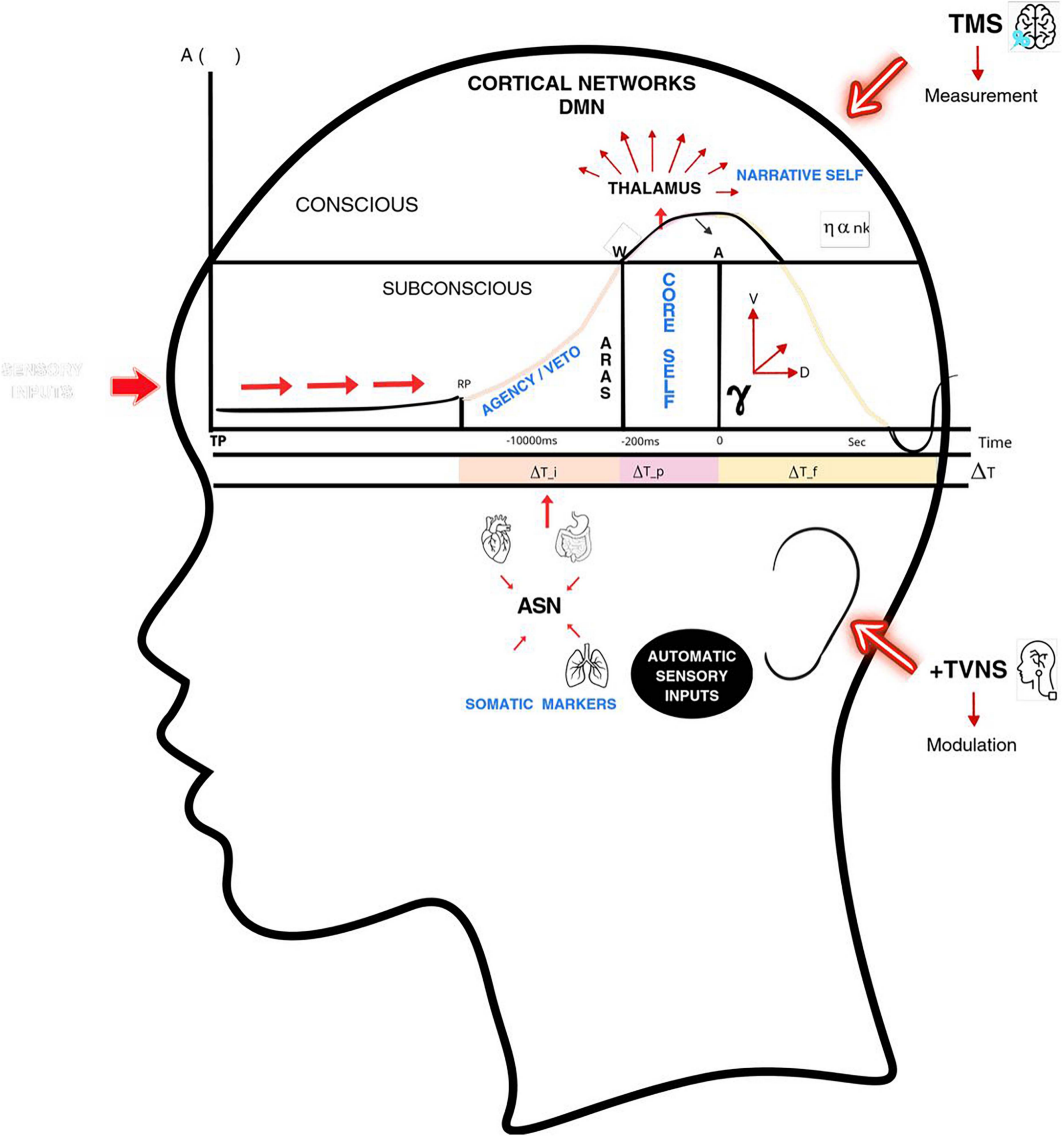
Operational temporal architecture of the Emergent Flow Theory (EFT). Schematic representation of EFT as a hierarchical model in which conscious processing depends on temporally constrained integration. Sensory inputs and autonomic–interoceptive signals converge across mesoscopic windows (ΔT^I^: preparatory/autonomic, spanning ∼800 ms before conscious intention; ΔT_p_: perceptual access, –200 to 0 ms; ΔT^f^: phenomenological/narrative continuity, 0 to several seconds). Basal coherence (γ) modulates the system’s readiness for integration, operationally indexed by heart rate variability (HRV) and serving as a temporal gain parameter–not a direct generator of conscious content–for the downstream perceptual and phenomenological windows. Cortical integration complexity (α_nk) reflects large-scale network interactions, including thalamocortical dynamics and Default Mode Network (DMN) engagement. The diagram also highlights candidate experimental readouts and perturbations: TMS–EEG measurements provide the Perturbational Complexity Index (PCI) as an empirical approximation of global integration capacity (), while transcutaneous vagus nerve stimulation (tVNS) serves as the primary modulation tool for γ. The ASN (Autonomic-Sensory Network) and ARAS (Ascending Reticular Activating System) are depicted as anatomical substrates linking peripheral interoceptive signals to cortical integration dynamics, consistent with EFT’s testable operational framework.

The original operational core of EFT focuses on three empirically supported mesoscopic temporal windows that characterize conscious processing in the central nervous system (CNS). These windows describe how information is integrated once it has entered neural circuits capable of supporting phenomenological access (see Figure 1).

ΔT^I^ (Autonomic or Preparatory Window): precedes conscious volition and corresponds to the integration of autonomic, interoceptive, and somatic signals that establish functional priors for action and perception. It resonates with the temporal dynamics of Libet’s Readiness Potential and spans hundreds of milliseconds to seconds. Processing at this level remains largely preconscious. This window corresponds to integration across the Autonomic-Sensory Network (ASN) and the Ascending Reticular Activating System (ARAS).
ΔT_p_ (Perceptual Window): represents the temporal interval required for sensory information to achieve global accessibility. It is associated with thalamocortical synchronization and the ignition of the global neuronal workspace (Dehaene and Changeux, 2011). Empirically, it corresponds to perceptual binding durations on the order of ∼100–300 ms and is reflected in components such as the P300.
ΔT^f^ (Phenomenological or Narrative Window): the broadest temporal window, supporting the subjective continuity of experience across moments. It is associated with Default Mode Network (DMN) activity, working memory, and autobiographical integration, allowing conscious contents to be linked across time into a coherent narrative flow.

### 2.3 A necessary extension: from neural input to the origin of coherence

While the ΔT*^I^*, ΔT^p^, and ΔT^f^ windows successfully describe the temporal dynamics of consciousness once information has reached the CNS, the current formulation leaves an important question unresolved: How does basal coherence (γ) itself emerge and consolidate in time?

In the operational core of EFT, γ functions as a modulatory input–an enabling condition for conscious integration. However, the framework remains incomplete if the temporal origin of γ is left unexplained. If temporal integration is a necessary condition for consciousness, this necessity cannot arise ex nihilo at the level of cortical neurons alone.

Basal coherence reflects the organism’s internal state–metabolic, autonomic, and interoceptive–which is itself the product of ongoing biological integration. Therefore, the requirement for a finite temporal window cannot be exclusive to neural systems but must extend to more fundamental biological levels, where information about the organism’s internal condition is first consolidated.

This motivates a necessary extension of EFT: the Minimum Biological Temporal Window (ΔT_b). ΔT_b is defined as the minimal duration required for non-neuronal biological systems–at the cellular and molecular level–to integrate stimuli into a stable informational state (e.g., cellular memory or state-dependent responsiveness). This extension does not challenge the operational core of EFT. Instead, it grounds that core in its most basic biological foundations.

## 3 Memory takes time: cellular evidence for temporal integration without neurons

### 3.1 Memory as the fundamental integrative process

The capacity to consolidate past information into a stable and retrievable state–commonly referred to as memory–is not an invention exclusive to the nervous system, but a fundamental requirement of any adaptive biological entity. From an evolutionary perspective, organisms that cannot bind information across time cannot learn from previous encounters, anticipate future states, or adapt their behavior based on experience. In this foundational sense, memory represents the most basic form of temporal integration in living systems.

This perspective shifts the analysis of consciousness from a question of where integration occurs (e.g., cortical columns, thalamocortical loops) to when it becomes physically possible. If memory–defined operationally as the time-dependent consolidation of information into a stable molecular or structural state–requires specific temporal windows even in single cells, then temporal integration is not a neural innovation but a biological constraint that predates and enables conscious processing.

Within EFT, conscious experience emerges from hierarchical temporal integration across three established windows: interoceptive (ΔT^I^), perceptual (ΔT_p_), and phenomenological (ΔT^f^). However, these windows presuppose an underlying biological system capable of registering and consolidating information in the first place. We designate the foundational cellular constraint as ΔT_b and argue that it constitutes the basal temporal substrate upon which all higher-order integrative processes are constructed.

Throughout this and the following sections, established empirical findings are distinguished from EFT-specific integrative interpretations. Where direct evidence is not yet available, claims are presented as biologically plausible hypotheses rather than established causal facts.

### 3.2 The Kukushkin paradigm: temporal discrimination in non-neuronal cells

The most compelling empirical evidence for cellular temporal integration comes from the work of Kukushkin and colleagues, who demonstrated that non-neuronal mammalian cells possess the capacity to discriminate temporal patterns and form memories based on stimulus spacing (Kukushkin and Carew, 2017; Kukushkin et al., 2024).

#### 3.2.1 The spaced learning effect at the cellular level

The spacing effect–the phenomenon whereby information presented in temporally spaced intervals is consolidated more effectively than information presented in a single continuous (massed) exposure–has long been documented in behavioral neuroscience. Kukushkin et al. (2024) extended this principle to human non-neuronal cells, including kidney cells and astrocytes.

When exposed to repeated chemical stimuli, these cells exhibited differential activation of the CREB/ERK molecular cascade–a well-characterized pathway for long-term memory consolidation–depending on the temporal spacing of stimulation. Cells exposed to spaced stimuli (separated by minutes) activated memory-associated genes, whereas cells exposed to massed stimuli (with no spacing) did not.

These findings carry three critical implications for the EFT framework: (1) memory is not neural-specific–the capacity to integrate information across time exists in cells without synapses; (2) temporal discrimination is molecular–cells effectively measure time through biochemical dynamics, requiring a finite temporal window (ΔT_b); (3) memory requires a minimum duration–below a critical interval, information cannot be consolidated into a stable cellular state.

In EFT, autonomic coherence is not treated as a generator of cortical activity, but rather as a modulatory condition that influences whether cortical integration can stabilize into conscious-access states.

#### 3.2.2 Mechanisms of temporal gating

The molecular basis of ΔT_b involves time-dependent biochemical integration. The ERK/CREB pathway–central to long-term potentiation (LTP) in neurons–operates through cascades of phosphorylation events that unfold over seconds to minutes and exhibit intrinsic temporal sensitivity. Stimuli must be sufficiently separated to allow partial system recovery, enabling the additive strengthening of intracellular signals required to activate memory-associated genes.

The functional outcome of this process is stable: cells that undergo spaced learning exhibit persistent changes in gene expression and altered responsiveness to future stimuli. In operational terms, the cell has encoded its past state–it has functionally remembered.

### 3.3 Operational definition of ΔT_b and critical clarification

Formal definition – ΔT_b: the minimum temporal interval required for a single cell to consolidate information from repeated stimuli into a stable molecular state (memory), characterized by sustained activation of plasticity-associated genes (e.g., CREB, c-Fos) and measurable changes in cellular response properties.

Key properties of ΔT_b: Duration: seconds to minutes (governed by biochemical kinetics). Mechanism: protein kinase cascades and transcriptional delays. Universality: present in both neuronal and non-neuronal cells. Functional output: determines stable memory consolidation versus transient activation.

Critical clarification: ΔT_b ≠ phenomenological consciousness. ΔT_b does not imply phenomenological consciousness at the cellular level. It represents a necessary but not sufficient condition for conscious processing–the bedrock upon which higher-level integrative architectures are constructed. The relationship is hierarchical: conscious systems inherit temporal constraints from their constituent biological processes without inheriting phenomenology itself.

### 3.4 Bridging ΔT_b to organismic coherence (γ): plausible mechanisms

The central conceptual step for EFT is mapping cellular temporal integration (ΔT_b) onto the emergent basal coherence (γ) that modulates conscious processing. We propose two complementary and testable routes by which aggregated cellular memory may contribute to γ.

*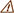 Tier-2: The pathways described in Sections 3.4.1–3.4.3 are classified as Tier-2: mechanistically plausible based on established physiology, but requiring empirical validation within the EFT framework*.

#### 3.4.1 Homeostatic baseline setting

Billions of cells across visceral organs, immune tissues, and glial networks operate with ΔT_b-dependent plasticity. The cumulative metabolic and bioelectrical state of these distributed cellular memories is proposed to establish a stable homeostatic landscape that the autonomic nervous system reads as interoceptive coherence (γ). In EFT terms, the integrated state of cellular memory is proposed to contribute to organism-level basal coherence (γ). This suggests a compositional hierarchy in which global coherence may inherit constraints from distributed cellular integration.

#### 3.4.2 Neuromodulatory feedback via the vagus nerve

Cellular states governed by ΔT_b may influence vagal afference. Peripheral cellular activity–such as cytokine release from immune cells or metabolic signaling from visceral tissues–is consistent with activation of vagal afferents, which relay integrated information to brainstem nuclei (e.g., the nucleus tractus solitarius, NTS). These nuclei, in turn, regulate autonomic output, influencing heart rate variability (HRV), the primary proxy for γ.

Critically, a distinction must be drawn between the timescale of cellular memory consolidation (ΔT_b, minutes) and the timescale of cellular signaling (milliseconds). While ΔT_b governs the establishment of stable molecular set-points, moment-to-moment bioelectric and chemical signals fluctuate rapidly around these set-points and are continuously integrated by the autonomic nervous system. Thus, ΔT_b does not gate individual signals but is proposed to influence the organism’s baseline autonomic coherence by establishing the physiological landscape within which signaling occurs.

#### 3.4.3 Long-term baseline constraints: a proposed multi-timescale coupling mechanism

The cellular memory mechanisms characterized by Kukushkin and colleagues operate at timescales of minutes to hours (ΔT_b). While this timescale differs substantially from the millisecond-to-second windows of autonomic coherence (γ), converging evidence from epigenetic regulation, allostatic load, and bioelectric physiology suggests a biologically plausible multi-timescale coupling mechanism linking sustained cellular integration to baseline autonomic regulation.

Nestler (2014) demonstrated that chronic patterns of neural activity produce durable epigenetic modifications–including DNA methylation, histone acetylation, and chromatin remodeling–in stress-responsive circuits, establishing molecular set-points that constrain future cellular responsiveness. McEwen (2007) extended this framework through the concept of allostatic load: the cumulative biological cost of sustained deviation from optimal regulatory states. Chronic dysregulation of autonomic tone produces measurable structural changes in hippocampal, amygdalar, and prefrontal circuits (McEwen and Wingfield, 2003). Levin (2019) provided a complementary perspective through bioelectric signaling, demonstrating that stable voltage gradients across tissues may function as distributed memory systems capable of coordinating multicellular behavior.

Within the EFT framework, we propose that the long-run distribution of γ values–reflecting sustained patterns of autonomic regulation over days, weeks, and months–may drive ΔT_b-dependent epigenetic and bioelectric changes across vagal-innervated tissues. These accumulated cellular memories would, in turn, establish the physiological baseline around which moment-to-moment γ fluctuations occur. This represents a proposed bidirectional coupling mechanism rather than a demonstrated causal pathway. It is classified as Tier-2: mechanistically plausible based on established physiology across multiple domains, empirically testable, but requiring direct validation within the EFT framework.

### 3.5 Experimental validation of multi-scale framework

The ΔT_b framework generates specific, falsifiable predictions, summarized in Table 2. Prediction 3.1: replication of cellular spacing effects across diverse human cell types to establish ΔT_b as an invariant biological principle. Prediction 3.2: correlated modulation of cellular ΔT_b markers and autonomic basal coherence (γ). Prediction 3.3: top-down modulation of ΔT_b markers. Conscious states (e.g., mindfulness meditation) should induce measurable changes in immune or glial CREB/c-Fos expression that correlate with enhanced γ, demonstrating bidirectional coupling.

**TABLE 2:** Empirical predictions and proposed methodologies.

Prediction	Method	Expected outcome	Tier
Replication of cellular ΔT_b	Cell culture + spaced vs. massed stimulation protocols	CREB/ERK activation depends on interstimulus interval (ISI)	Tier-2
ΔT_b →γ mechanistic link	CREB pathway inhibitors + HRV monitoring in vagal-innervated tissue preparations	Reduced γ (HRV) correlates with impaired cellular memory markers	Tier-2
Top-down modulation of ΔT_b	Mindfulness meditation training + immune cell CREB/c-Fos assays	Increased γ predicts altered expression in peripheral cells	Tier-2
Perturbational complexity (α_nk) modulation via γ	TMS + EEG Perturbational Complexity Index (PCI)	Modulation of γ (via tVNS) predicts changes in cortical complexity	Tier-1

All predictions can be tested with existing neurophysiological and molecular biology techniques. Tier-1, empirically supported component ready for direct test; Tier-2, mechanistically plausible, requires validation within EFT context. tVNS, transcutaneous vagus nerve stimulation; HRV, heart rate variability; PCI, Perturbational Complexity Index.

### 3.6 Section summary

The work of Kukushkin and colleagues demonstrates that non-neuronal cells form memories governed by a biochemical temporal constraint (ΔT_b). This requirement for time represents the most fundamental layer of information consolidation in living systems. Within EFT, ΔT_b is proposed to plausibly contribute to emergent homeostatic coherence (γ), which in turn enables higher-level temporal integration associated with conscious processing. ΔT_b is the temporal foundation–not the experience itself.

## 4 Bioelectricity and basal cognition: a mechanistic proposal bridging cellular ΔT_b and organismic γ

### 4.1 The problem of scale: from molecular memory to organismic coherence

The evidence presented in Section “3 Memory takes time: cellular evidence for temporal integration without neurons” establishes that individual cells possess temporal integration capacities governed by ΔT_b. However, a critical mechanistic question remains: how do billions of distributed cellular integration processes, each operating with its own ΔT_b, collectively contribute to the unified basal coherence (γ) that modulates conscious processing at the organismic level?

This is not merely a problem of scale, but a problem of translation. Molecular memory mechanisms–such as CREB/ERK activation, phosphorylation cascades, and gene transcription–operate in biochemical currencies (protein concentrations, ion-channel expression, transcriptional delays). In contrast, organismic basal coherence (γ) is expressed through large-scale autonomic synchronization, including heart rate variability, respiratory coupling, and neuromodulatory tone.

We propose that bioelectricity–the collective voltage dynamics of cells, tissues, and organ systems–may provide such a translational medium. Recent work on bioelectric computation and basal cognition demonstrates that living tissues use voltage gradients not only for rapid signaling but also as persistent informational substrates capable of storing, integrating, and transmitting adaptive states across biological scales (Fields and Levin, 2020; Levin, 2024).

### 4.2 Bioelectricity as a multi-scale informational medium

#### 4.2.1 Beyond neural action potentials: tonic bioelectric states

Classical neuroscience emphasizes phasic bioelectricity, namely the rapid, all-or-none action potentials of excitable cells. However, accumulating evidence highlights the functional importance of tonic bioelectricity: slow, graded voltage dynamics present in all living cells, including non-excitable tissues (Levin, 2014). Key properties include universality (all living cells maintain resting membrane potentials), information storage (stable voltage states encode functional cellular identities), spatial coupling (gap junctions link cells into bioelectric networks), and temporal integration (bioelectric states exhibit memory-like behavior as past stimuli modify ion-channel expression and future responsiveness).

Crucially, these voltage dynamics operate on timescales compatible with ΔT_b. Changes in ion-channel trafficking and membrane polarization consolidate over minutes to hours, aligning with the biochemical temporal windows required for cellular memory formation described in Section “3 Memory takes time: cellular evidence for temporal integration without neurons.”

#### 4.2.2 Bioelectric patterning and functional control

Bioelectric patterns may function as distributed control systems capable of coordinating tissue-level behavior without centralized oversight (Levin, 2024). Illustrative examples include planarian head–tail polarity that is consistent with dependence on bioelectric gradients across tissues (Durant et al., 2017), tumor suppression via restoration of normal polarization states (Chernet and Levin, 2013), and developmental patterning through imposed bioelectric gradients (Pai et al., 2012). EFT proposes that developmental bioelectric organization may leave durable constraints on later autonomic and neural integration.

These findings illustrate–rather than prove–that bioelectricity can integrate distributed cellular states into coherent tissue-level outcomes, providing a plausible mechanistic route by which ΔT_b-dependent cellular memories may influence higher-order physiological coherence. This hypothesis is consistent with the Tier-2 status of the proposed ΔT_b →γ pathway.

### 4.3 Bioelectricity and the autonomic nervous system: the γ interface

The autonomic nervous system (ANS), coordinated by brainstem nuclei such as the nucleus tractus solitarius (NTS), parabrachial nucleus (PBN), locus coeruleus (LC), and raphe nuclei, serves as the primary integrator of visceral information. In EFT, basal coherence (γ) represents the degree of autonomic synchronization that enables higher-order temporal integration.

#### 4.3.1 Vagal afferents as integrative sensors

Approximately 80% of vagal fibers are afferent, conveying integrated visceral information to the brainstem (Berthoud and Neuhuber, 2000). These fibers respond to mechanical, chemical, and electrophysiological tissue states, encoding aggregate tissue conditions rather than isolated molecular events. Representative examples include the inflammatory reflex (Tracey, 2002), gut–brain axis metabolic integration (Mayer, 2011), and cardiac interoception shaping autonomic regulation and emotional experience (Critchley and Garfinkel, 2017).

Within EFT, these pathways suggest that γ may reflect the integrated bioelectric and metabolic state of peripheral tissues shaped by cumulative cellular temporal integration.

#### 4.3.2 From vagal input to basal coherence

Vagal afferents converge on the NTS, which projects to neuromodulatory systems governing arousal, affect, and temporal binding. Through these pathways, distributed cellular states may be translated into organismic basal coherence. The proposed functional pathway is:

*ΔT_b-dependent cellular integration → bioelectric state shifts → vagal afferent signaling → brainstem integration (NTS, PBN, LC) →γ modulation*
*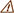 Tier-2: This pathway is classified Tier-2: grounded in established biological principles but requiring empirical demonstration within the EFT context.*

### 4.4 Basal cognition: a non-phenomenological definition

The concept of basal cognition formalizes the observation that goal-directed regulation, learning, and memory can occur in biological systems lacking nervous systems (Levin, 2024; Lyon et al., 2021). Throughout this section, cognition at the cellular or tissue level refers strictly to time-dependent state integration and adaptive regulation, not to phenomenological awareness. In its most minimal operational form: Cognition = Temporal integration + adaptive regulation. Within EFT, ΔT_b captures the temporal component of this definition.

The term “basal” appears in two related but distinct contexts: (1) basal coherence (γ) refers to organismic baseline physiological synchronization, whereas (2) basal cognition refers to minimal adaptive regulation in non-neural systems. Both share the sense of “foundational” but operate at different organizational levels.

### 4.5 The Principle of Temporal Invariance Across Scales

We articulate the Principle of Temporal Invariance, a unifying heuristic within EFT: the functional output of temporal integration at one level of biological organization acts as a basal coherence constraint for integration at the next level. At the cellular level, ΔT_b governs biochemical integration. At the organismic level, the aggregate output of cellular integration is proposed to contribute to γ, which modulates neural integration windows (ΔT^I^, ΔT_p_, ΔT^f^). This framing yields a testable implication: perturbations at the ΔT_b level should propagate upward to influence γ and, indirectly, higher-order integration dynamics.

### 4.6 Bidirectional causality and top-down modulation

The hierarchy described above is not unidirectional. Conscious and emotional states may modulate cellular integration through autonomic pathways, establishing a closed causal loop. Evidence consistent with this view includes associations between mindfulness practices and gene expression changes (Kaliman et al., 2014), stress-related alterations in cellular aging markers (Epel et al., 2004), and placebo effects mediated by autonomic regulation (Benedetti, 2014). Within EFT, these findings are consistent with the hypothesis that conscious states may reshape basal coherence (γ), which in turn may influence ΔT_b-dependent cellular processes, completing a biologically plausible feedback loop.

### 4.7 Section summary

This section proposes bioelectricity as a mechanistic bridge between cellular temporal integration (ΔT_b) and organismic basal coherence (γ). Through tonic voltage dynamics, gap-junction coupling, and vagal–autonomic integration, distributed cellular memory states may be translated into a coherent physiological landscape that modulates conscious processing. This proposal is grounded in established findings from bioelectric physiology, interoceptive neuroscience, and autonomic regulation, unified by the Principle of Temporal Invariance Across Scales. Taken together, these findings support a biologically plausible integrative interpretation, but not yet a demonstrated EFT-specific causal pathway. EFT therefore treats these mechanisms as convergent evidence supporting the model’s architecture rather than as definitive proof of its full causal structure.

## 5 Synthesis: the unified temporal architecture of consciousness

### 5.1 Integration across hierarchical scales

This section synthesizes the temporal components developed in the previous sections into a unified framework. EFT concludes that consciousness is not a localized event but a temporally distributed process emerging from hierarchical integration across multiple time windows (Figure 1 and Tables 3, 4). Specifically, conscious experience arises from the seamless coordination of four primary temporal scales:

ΔT_b (Bioelectrical/Biochemical Window): the foundational consolidation of cellular information through molecular cascades such as ERK/CREB activation, governing non-neuronal and neuronal cellular memory. (Tier-2: proposed mechanistic link to γ requires validation.)
ΔT^I^ (Integrative/Autonomic Window): a preparatory window spanning approximately 800 ms before reported conscious intention (−1000 to −200 ms), during which autonomic, interoceptive, and premotor signals are integrated into functional priors for perception and action.
ΔT_p_ (Perceptual Window): the access window (−200 to 0 ms) in which thalamocortical integration and large-scale cortical recruitment enable perceptual awareness and conscious accessibility.
ΔT^f^ (Phenomenological Window): a supra-second window supporting narrative continuity and egoic self-representation, associated with Default Mode Network activity.

**TABLE 3:** Operational temporal windows in EFT (empirically testable).

Window	Duration	Neural substrate	Experimental marker	Verification method
ΔT^I^	−1000 to −200 ms	ASN → ARAS	Readiness Potential (RP)	EEG + HRV + tVNS modulation
ΔT_p_	−200 to 0 ms	ARAS → thalamocortical networks	P300, global ignition	TMS-EEG (PCI)
ΔT^f^	0 to 3000 ms	Thalamus → DMN	BOLD signal coherence	fMRI of DMN networks

Each temporal window (ΔT) represents a necessary constraint for conscious integration according to the EFT heuristic integration relation: = γ⋅ n ⋅α_nk ⋅ΔT. γ (basal coherence) emerges from autonomic integration during ΔT^I^; α_nk (cortical complexity) is measured via TMS-EEG during ΔT_p_; ΔT^f^ supports narrative continuity through DMN coherence. ASN, Autonomic-Sensory Network; ARAS, Ascending Reticular Activating System; HRV, heart rate variability; tVNS, transcutaneous vagus nerve stimulation; PCI, Perturbational Complexity Index; DMN, Default Mode Network.

**TABLE 4:** Emergent Flow Theory (EFT) in relation to contemporary theories of consciousness.

Theory	What integrates	When integrates	Autonomic regulation	How EFT complements
Global neuronal workspace (GNW)	Long-range workspace neurons enabling broadcast	Ignition event (∼300 ms)	Arousal acknowledged; not parametrized	Formalizes neuromodulatory preconditions (γ) as a quantifiable parameter; specifies autonomic conditions for ignition probability
Integrated Information Theory (IIT)	Cause-effect structure (Φ)	Atemporal structural property	Not addressed	Adds temporal dimension: specifies how Φ (indexed by PCI) may vary with autonomic state across integration windows
Predictive processing/FEP	Hierarchical prediction errors	Continuous Bayesian updating	Precision weighting (implicit)	Specifies γ as a physiological substrate for precision weighting; provides measurable HRV proxy for precision parameter
Emergent Flow Theory (EFT)	Nested temporal windows: ΔT_b →ΔT^I^→ΔT_p_ →ΔT^f^	Hierarchical, from cellular (min) to phenomenological (sec)	Central parameter (γ): quantifiable via HRV; manipulable via tVNS	Addresses temporal-autonomic dimension not formalized in GNW, IIT, or PP/FEP; generates falsifiable predictions (Table 2)

EFT is proposed as complementary to, rather than competing with, GNW, IIT, and PP/FEP. HRV, heart rate variability; PCI, Perturbational Complexity Index; tVNS, transcutaneous vagus nerve stimulation; FEP, Free Energy Principle.

These windows are not independent stages but interdependent temporal constraints that together define the organism’s capacity to bind past, present, and future information into a coherent conscious flow.

### 5.2 Reinterpreting the Libet paradigm through EFT

Within this temporal architecture, the classical findings of Libet are recontextualized not as evidence against agency, but as a map of necessary integration delays. The interval between the onset of the Readiness Potential (RP) and the reported moment of conscious intention (W) may reflect the time required for basal cellular and autonomic information to reach a sufficient level of functional integration complexity (α_nk) for phenomenological access.

## 6 Discussion and outlook

### 6.1 Positioning EFT as complementary to existing frameworks

Emergent Flow Theory is positioned as complementary to–rather than competing with–dominant frameworks in consciousness science (Table 4). Each framework addresses different dimensions of the integration problem, and EFT’s contribution lies in formalizing an aspect that existing theories do not formalize in the specific autonomic-temporal manner proposed here.

The global neuronal workspace theory (GNW; Dehaene and Changeux, 2011) specifies the cortical architecture through which conscious broadcast occurs via ignition of fronto-parietal networks. EFT proposes that autonomic coherence (γ) functions as a physiological parameter that modulates the probability of ignition, complementing GNW by adding a quantifiable autonomic dimension.

Integrated Information Theory (IIT; Tononi et al., 2016) characterizes the structural conditions for high integrated information (Φ), operationally indexed by PCI. EFT adds a temporal perspective: How does Φ–or its empirical proxy PCI–vary dynamically as a function of autonomic state, across the cardiac cycle, or following autonomic perturbation?

The Predictive Processing framework and Free Energy Principle (PP/FEP; Friston, 2010) formalize precision weighting–the modulation of prediction error gain by neuromodulators. EFT is consistent with this framework and proposes autonomic coherence (γ), indexed by HRV, as a variable governing the precision of ascending interoceptive signals, specifying a concrete physiological substrate for precision weighting.

In summary, EFT addresses a dimension of conscious integration–temporal architecture modulated by autonomic state–that GNW, IIT, and PP/FEP do not formalize in the specific autonomic-temporal manner proposed here. Rather than replacing these frameworks, EFT provides a complementary physiological layer that may enhance their empirical completeness.

### 6.2 Temporal integration as empirically tractable entry point: acknowledging spatial constraints

An important conceptual scope limitation should be acknowledged explicitly. EFT focuses on temporal integration as an empirically tractable entry point for investigating conscious processing, without claiming that temporal structure alone exhausts the full organizational basis of consciousness.

Many biological mechanisms invoked in EFT–bioelectric signaling, vagal afference, thalamocortical synchronization, and cortical network dynamics–are inherently spatially structured. Bioelectric gradients propagate across specific tissue topologies (Levin, 2014); vagal afferents originate from spatially distributed visceral receptors and converge on anatomically localized brainstem nuclei; cortical integration reflects structured patterns of long-range connectivity. From this perspective, EFT’s temporal hierarchy may represent a tractable projection of a higher-dimensional spatiotemporal system. Northoff and Huang (2017) proposed a temporo-spatial theory of consciousness (TTC) emphasizing the joint contribution of temporal scales and spatial topographies. Future integration of EFT’s autonomic-temporal framework with TTC’s spatial emphasis could yield a more comprehensive account (Hasson et al., 2008).

Emergent Flow Theory therefore treats temporal integration as a necessary and experimentally tractable axis of conscious processing, without claiming that temporal structure alone exhausts the full organizational basis of consciousness. This limitation does not diminish EFT’s contribution, and spatiotemporal integration represents a natural extension of, rather than a refutation of, the approach initiated here.

Speculative extensions toward quantum-scale temporal constraints (ΔT_q) are discussed in Supplementary Material 1 as a theoretical research horizon and are not part of the core claims, predictions, or evidentiary basis of the present manuscript.

### 6.3 A cross-scale temporal constraint: a recursive organizational logic

A central contribution of this work is the formulation of a cross-scale temporal constraint: integrative biological functions require a finite minimum temporal window (ΔT) for information to become stable, functionally accessible, and behaviorally relevant. By linking evidence for temporally gated integration at the cellular level (ΔT_b) with established mesoscopic windows of perceptual access (ΔT^p^) and phenomenological continuity (ΔT^f^), EFT motivates a recursive organizational logic in which time-limited integration reappears across levels of biological organization.

Working Principle (Cross-Scale Temporal Constraint). The functional output of temporally constrained integration at one level of organization can act as a boundary condition–a coherence constraint (γ)–for integration at the next level.

Critically, this principle does not entail phenomenological continuity across scales. Instead, it proposes functional continuity: time-dependent consolidation at lower levels shapes the stability and availability of higher-level integrative dynamics. ΔT_b is proposed as a biologically grounded extension supported by existing evidence in cellular memory, while speculative extensions beyond the operational framework are reserved for Supplementary Material 1.

Under this framework, consciousness is modeled not as a categorical discontinuity from biological regulation, but as a high-order amplification of temporally constrained integration–supported by physiological coherence and expressed as phenomenological accessibility.

### 6.4 Clinical and translational implications (hypothesis-generating)

Emergent Flow Theory suggests that changes in basal coherence (γ) may causally shape the system’s capacity for complex cortical integration (α_nk) within perceptual and phenomenological time windows. From this perspective, conditions such as PTSD, addiction, depression, and obsessive–compulsive disorder may involve fragmentation or maladaptive tuning of temporal integration windows (ΔT*^I^*, ΔT_p_, ΔT^f^), potentially linked to dysregulated autonomic–interoceptive coupling (γ). Neuromodulation approaches such as transcutaneous vagus nerve stimulation (tVNS) and transcranial magnetic stimulation (TMS) may be conceptualized as candidate interventions that perturb and potentially stabilize basal coherence and cortical integration dynamics.

Importantly, these implications are hypothesis-generating. EFT does not propose immediate clinical prescriptions. Instead, it motivates testable translational questions–for example: whether experimentally increasing γ (e.g., via tVNS protocols) produces reliable, quantifiable shifts in α_nk (e.g., PCI) and in behavioral proxies of temporal integration (see Table 2).

### 6.5 Limitations and near-term validation priorities

While this framework integrates cellular, autonomic, and cortical processes into a unified temporal architecture, several limitations should be explicitly acknowledged.

First, the proposed link between cellular ΔT_b and organismic γ is mechanistically plausible but not yet empirically established. A decisive test would require concurrent measurement of (i) cellular consolidation markers (e.g., CREB/ERK dynamics) in peripheral, vagal-innervated tissues, (ii) autonomic coherence indices (e.g., HRV and cardiorespiratory coupling), and (iii) cortical integration complexity (α_nk) using perturbational measures (e.g., TMS–EEG-derived PCI). Such multimodal studies are feasible but resource-intensive.

Second, the cross-scale temporal constraint is advanced as a working principle rather than a mathematically derived law. The recursive use of the EFT heuristic integration relation across scales is a functional analogy intended to organize testable hypotheses. A formal derivation from first-principles biophysics remains an open theoretical objective.

Third, EFT focuses on temporal integration as an empirically tractable entry point, recognizing that spatial organization–though acknowledged in discussions of bioelectric gradients, vagal pathways, and cortical networks–requires further formal development.

Finally, EFT is best interpreted at present as a structured hypothesis space defining what to measure, when to measure it, and how to link levels in experimental designs. It is explicitly a Tier-2 theoretical framework at its mechanistic core: its claims are grounded in established empirical literature, but the integrated pathway from ΔT_b through γ to cortical complexity has not yet been tested as a unified system. The framework generates falsifiable predictions (Table 2) designed to elevate specific components from Tier-2 (plausible) to Tier-1 (validated).

## 7 Conclusion

Consciousness is not merely a state; within EFT, it can be modeled as a temporally constrained architecture of integration. By linking non-neuronal cellular consolidation (ΔT_b) and bioelectric signaling to autonomic coherence (γ) and to established cortical-perceptual integration windows (ΔT^I^, ΔT_p_, ΔT^f^), the Emergent Flow Theory provides a unified framework that connects biological foundations with phenomenological accessibility.

Within this view, the central question is not only where consciousness occurs, but how time enables biological systems to stabilize, integrate, and render information functionally accessible across levels of organization. EFT reframes conscious processing as an emergent outcome of coordinated temporal integration–rooted in cellular biology, expressed through physiology, and realized as subjective experience.

By positioning autonomic coherence as a temporal gain parameter rather than a direct generator of conscious content, EFT offers a biologically grounded and empirically testable complement to existing theories of conscious integration.

## Data Availability

The original contributions presented in the study are included in the article/Supplementary material, further inquiries can be directed to the corresponding author/s.
