# Supplementary material for: Consciousness emerges from temporal integration across biological scales: from cellular memory to phenomenological experience
Source: Front Integr Neurosci. 2026 May 12;20:1772467. doi: 10.3389/fnint.2026.1772467 (PMC13201435; doi:10.3389/fnint.2026.1772467)
Supplement: Supplementary file 1 [file Data_Sheet_1.pdf]

SUPPLEMENTARY MATERIAL S1

Consciousness Emerges from Temporal Integration Across Biological Scales

Alfredo López Parra

CRITICAL PREFATORY DISCLAIMERS — PLEASE READ BEFORE PROCEEDINGThe material below is classified as Tier-3: speculative theoretical exploration.WHAT THIS MATERIAL IS NOT: (1) NOT required for the falsifiability of the main EFT framework; (2) NOT part of the empirical predictions in Table 4 of the main manuscript; (3) NOT part of the defense of the main manuscript under peer review; (4) NOT supported by current direct experimental evidence; (5) NOT currently testable with available methods in conscious biological systems; (6) NOT a claim about the substrate of consciousness.WHAT THIS MATERIAL IS: A disciplined theoretical research horizon exploring potential extensions of EFT's temporal hierarchy toward quantum-scale timescales, presented with full epistemic transparency. It is included for intellectual completeness and strictly separated from the operational framework.IMPORTANT: Readers and reviewers are strongly encouraged to evaluate the main manuscript entirely independently of this speculative content. The acceptance or rejection of ΔT_q does not affect the validity, falsifiability, or scientific merit of EFT's core operational framework.

S1. Quantum-Scale Temporal Constraints: The ΔT_q Hypothesis (Tier-3: Explicitly Speculative)

> Tier-3 classification. This section presents a speculative theoretical extension. It contains no empirical claims, generates no falsifiable predictions distinguishable from classical alternatives with current methods, and does not contribute to the empirical defense of EFT.

S1.1. Conceptual Motivation

The operational EFT framework spans from cellular consolidation timescales (ΔT_b: seconds to minutes) through autonomic-cortical windows (ΔT_i, ΔT□, ΔT_{f}: milliseconds to seconds). A logically consistent extrapolation of the cross-scale temporal constraint principle asks whether analogous temporal constraints apply at even more fundamental physical levels, where quantum coherence sets limits on information processing at the molecular scale.

We denote this hypothetical timescale as ΔT_q, defined as the minimal duration over which quantum coherence in a biological substrate could persist before environmental decoherence eliminates phase-sensitive information. In this view, ΔT_q would not be invoked as the substrate of consciousness. Rather, it would be framed as a physical constraint that, where quantum coherence may set limits on information processing at the molecular scale, might—under specific biological implementations—shape the reliability of microscopic informational dynamics that cascade upward into biochemical and physiological organization.

S1.2. Candidate Quantum Biological Phenomena

Several quantum biological phenomena have been proposed as candidates for functional coherence at biologically relevant timescales. Radical pair mechanisms: Spin-correlated radical pairs in cryptochrome photoreceptors exhibit coherence times on the order of microseconds, exceeding classical expectations in warm biological environments (Ritz et al., 2000). While functionally demonstrated in avian magnetoreception, extension to neural or conscious processing contexts is speculative.

Excitonic energy transfer: Long-lived quantum coherences have been reported under specific experimental conditions in photosynthetic complexes (Fleming et al., 2007), though the duration and functional significance of these coherences in physiologically realistic environments remains contested, and their applicability to neural systems has not been demonstrated.

Enzymatic tunneling: Proton and electron tunneling in enzymatic reactions may enhance catalytic efficiency, operating on femtosecond timescales. These processes are unlikely to be informationally relevant at scales connecting to cellular memory or autonomic coherence.

None of these phenomena have been demonstrated to causally influence cellular information consolidation (ΔT_b) or organismic autonomic coherence (γ). The distance between femtosecond quantum coherence and the millisecond-to-minute timescales of cellular and neural processing represents a many-orders-of-magnitude gap requiring independently validated bridging mechanisms at each level.

S1.3. Relationship to Orchestrated Objective Reduction (Orch OR)

The most developed quantum theory of consciousness is the Orchestrated Objective Reduction (Orch OR) hypothesis (Hameroff & Penrose, 1996), which proposes that quantum superpositions in microtubule tubulins undergo objective reduction at Planck-scale timescales, generating moments of proto-conscious experience. EFT's ΔT_q is conceptually distinct from Orch OR in the following respects: (1) EFT does not propose quantum superposition as the substrate of consciousness. (2) EFT does not invoke the Penrose objective reduction criterion or Platonic mathematical forms. The theoretical basis is classical decoherence theory applied to warm biological environments. (3) ΔT_q in EFT is presented as the lower boundary of a temporal hierarchy that is primarily classical, not as a causal mechanism generating conscious experience.

S1.4. What a ΔT_q Test Would Require

Testing any ΔT_q-related conjecture within EFT's framework would require demonstrating all of the following, none of which is currently feasible: (a) That quantum coherence times in specific biological molecules (in situ, at physiological temperatures and ionic conditions) correlate measurably with functionally relevant cellular states. (b) That perturbations of quantum coherence at the molecular level propagate upward to measurable changes in ΔT_b markers. (c) That such perturbations subsequently influence γ (HRV indices) and cortical complexity (PCI), closing the full cross-scale causal chain. This experimental program is beyond current technical capabilities for conscious biological systems.

S1.5. Why ΔT_q Is Retained as a Conceptual Horizon

Despite its speculative status, ΔT_q is retained in EFT's conceptual architecture for the following reason: the Principle of Temporal Invariance Across Scales—if valid—would apply without restriction across all organizational levels. Excluding the molecular quantum level entirely would risk introducing an arbitrary lower boundary to the temporal hierarchy. The honest position is to acknowledge the logical extension of the principle while maintaining full epistemic transparency about its speculative and currently untestable character.

S2. Epistemic Caveats and Current Limitations of ΔT_q

The ΔT_q hypothesis faces the following substantial and currently unresolved challenges: (1) No demonstrated mechanism linking femtosecond-to-picosecond quantum coherence to millisecond-to-minute neural and cellular integration has been experimentally established. The temporal gap spans approximately 9–15 orders of magnitude. (2) Decoherence in warm, wet neural tissue occurs orders of magnitude faster than proposed functional timescales. This concern is consistent with prior analyses suggesting that decoherence times in neural systems are likely far shorter than

neurophysiologically relevant processing windows (Tegmark, 2000). (3) No experimental paradigm currently exists that can test the causal role of quantum coherence in generating or modulating conscious processing, as distinct from classical thermodynamic explanations. (4) ΔT_q does not generate falsifiable predictions distinguishing quantum from classical mechanisms using methods available to EFT's current experimental program. (5) The reputational and scientific risks of conflating quantum speculation with EFT's empirically grounded core are acknowledged.

FINAL STATEMENT: For these reasons, ΔT_q is strictly speculative (Tier-3) and is NOT incorporated into EFT's empirical framework. It does not participate in the predictions in Table 4 of the main manuscript. Its inclusion here is solely as a transparent theoretical research horizon. The core EFT framework—spanning ΔT_b (Tier-2) through ΔT_i, ΔT□, and ΔT𝒻(Tier-1)—stands independently of these speculative extensions and should be evaluated entirely on its own merits.

Supplementary References

Fleming, G. R., Scholes, G. D., and Cheng, Y.-C. (2007). Quantum effects in biology. Proceedings of the National Academy of Sciences, 104(31), 12596–12601. https://doi.org/10.1073/pnas.0703739104

Hameroff, S., and Penrose, R. (1996). Orchestrated reduction of quantum coherence in brain microtubules: A model for consciousness. Mathematics and Computers in Simulation, 40(3–4), 453–480. https://doi.org/10.1016/0378-4754(96)80476-9

Ritz, T., Adem, S., and Schulten, K. (2000). A model for photoreceptor-based magnetoreception in birds. Biophysical Journal, 78(2), 707–718. https://doi.org/10.1016/S0006-3495(00)76629-X

Tegmark, M. (2000). Importance of quantum decoherence in brain processes. Physical Review E, 61(4), 4194–4206. https://doi.org/10.1103/PhysRevE.61.4194
